# Spontaneous Ovarian Hyperstimulation Syndrome in Natural Conception: A Case Report

**DOI:** 10.7759/cureus.83110

**Published:** 2025-04-28

**Authors:** Fatema Baqer, Njood Alsudairy

**Affiliations:** 1 College of Medicine, Jordan University of Science and Technology, Irbid, JOR; 2 Radiology, The Second Jeddah Health Cluster, Jeddah, SAU

**Keywords:** ascites, case report, early pregnancy, maternal health, natural pregnancy, ohss, ovarian enlargement, pleural effusion, spontaneous ovarian hyperstimulation syndrome, vascular permeability

## Abstract

Ovarian hyperstimulation syndrome (OHSS) is a serious condition usually associated with assisted reproductive technologies, but spontaneous OHSS occurring in natural pregnancies is a rare and often overlooked entity. We report the case of a 28-year-old woman, gravida 2 para 1, who presented in early pregnancy with progressive abdominal distension, dyspnea, and hemoconcentration without any history of fertility treatment. Imaging revealed bilateral multicystic ovarian enlargement, significant ascites, and small pleural effusions, while laboratory studies confirmed elevated endogenous human chorionic gonadotropin levels. She was diagnosed with moderate-to-severe spontaneous OHSS and was managed conservatively with intravenous fluids, correction of electrolyte imbalances, thromboprophylaxis, and ultrasound-guided therapeutic paracentesis, leading to gradual clinical improvement. The pregnancy remained viable, and the patient was discharged in stable condition with close follow-up. This case highlights the need for heightened clinical suspicion of spontaneous OHSS in early pregnancy, even in the absence of ovulation induction, as early diagnosis and supportive management are critical for optimizing maternal and fetal outcomes.

## Introduction

Ovarian hyperstimulation syndrome (OHSS) is a well-recognized iatrogenic complication of assisted reproductive technologies, particularly following ovulation induction with exogenous gonadotropins. It is characterized by ovarian enlargement, increased vascular permeability, fluid shifts into third spaces, and a hypercoagulable state [1-3]. Although OHSS is typically associated with fertility treatments, spontaneous cases occurring in natural pregnancies are rare and often under-recognized. Spontaneous OHSS is believed to be mediated by elevated levels of endogenous human chorionic gonadotropin (hCG), which stimulates ovarian vascular endothelial growth factor (VEGF) production, resulting in increased vascular permeability and the clinical manifestations of the syndrome [2,4]. Certain conditions associated with markedly elevated hCG levels, such as multiple gestations, molar pregnancies, or hypothyroidism, may predispose to spontaneous OHSS. Early recognition is critical, as severe cases can result in significant morbidity, including ascites, pleural effusions, electrolyte imbalances, thromboembolic events, and renal impairment. Diagnosis is primarily clinical, supported by imaging and laboratory studies, while management is largely supportive and focused on fluid balance, thromboprophylaxis, and symptom control [2,3]. We present a rare case of spontaneous OHSS occurring in a singleton spontaneous pregnancy, emphasizing the need for high clinical suspicion even in the absence of fertility treatment and discussing the diagnostic challenges, management strategies, and patient outcomes.

## Case presentation

A 28-year-old woman, gravida 2 para 1, presented to the emergency department with progressive abdominal distension, severe lower abdominal pain, nausea, and shortness of breath over the past five days. Her last menstrual period was approximately seven weeks prior to the presentation. She denied any history of assisted reproductive technology use, ovulation induction, or fertility treatment. Her previous pregnancy, two years ago, was uncomplicated and delivered at term via spontaneous vaginal delivery. She had no significant past medical history, surgical history, or family history of thromboembolic disorders or malignancy. She reported regular menstrual cycles prior to this pregnancy. She did not smoke, consume alcohol, or use illicit drugs. There was no history of prior ovarian cysts or gynecologic disorders.

Upon initial examination, the patient appeared in moderate respiratory distress, with tachypnea at 24 breaths per minute and oxygen saturation of 92% on room air. Her vital signs revealed a blood pressure of 110/65 mmHg, a pulse rate of 112 beats per minute, and a low-grade fever of 37.8°C. She had marked abdominal distension with diffuse tenderness, especially in the lower quadrants, and shifting dullness suggestive of ascites. There was no rebound tenderness or guarding. Bowel sounds were hypoactive. No peripheral edema was noted. The cardiopulmonary examination was significant for decreased breath sounds at both lung bases, suggesting a possible pleural effusion. The remainder of her physical examination was unremarkable.

Initial laboratory investigations revealed hemoconcentration with a hematocrit of 47% (normal: 36-44%), leukocytosis with a white blood cell count of 14,500/mm³, and mild electrolyte imbalances, including hyponatremia (serum sodium: 130 mmol/L) and hyperkalemia (serum potassium: 5.4 mmol/L). Serum creatinine was mildly elevated at 1.4 mg/dL. Liver function tests were within normal limits. Serum beta-hCG was 75,000 IU/L, consistent with early pregnancy. Coagulation studies showed elevated D-dimer levels but normal prothrombin time and activated partial thromboplastin time.

**Figure 1:**
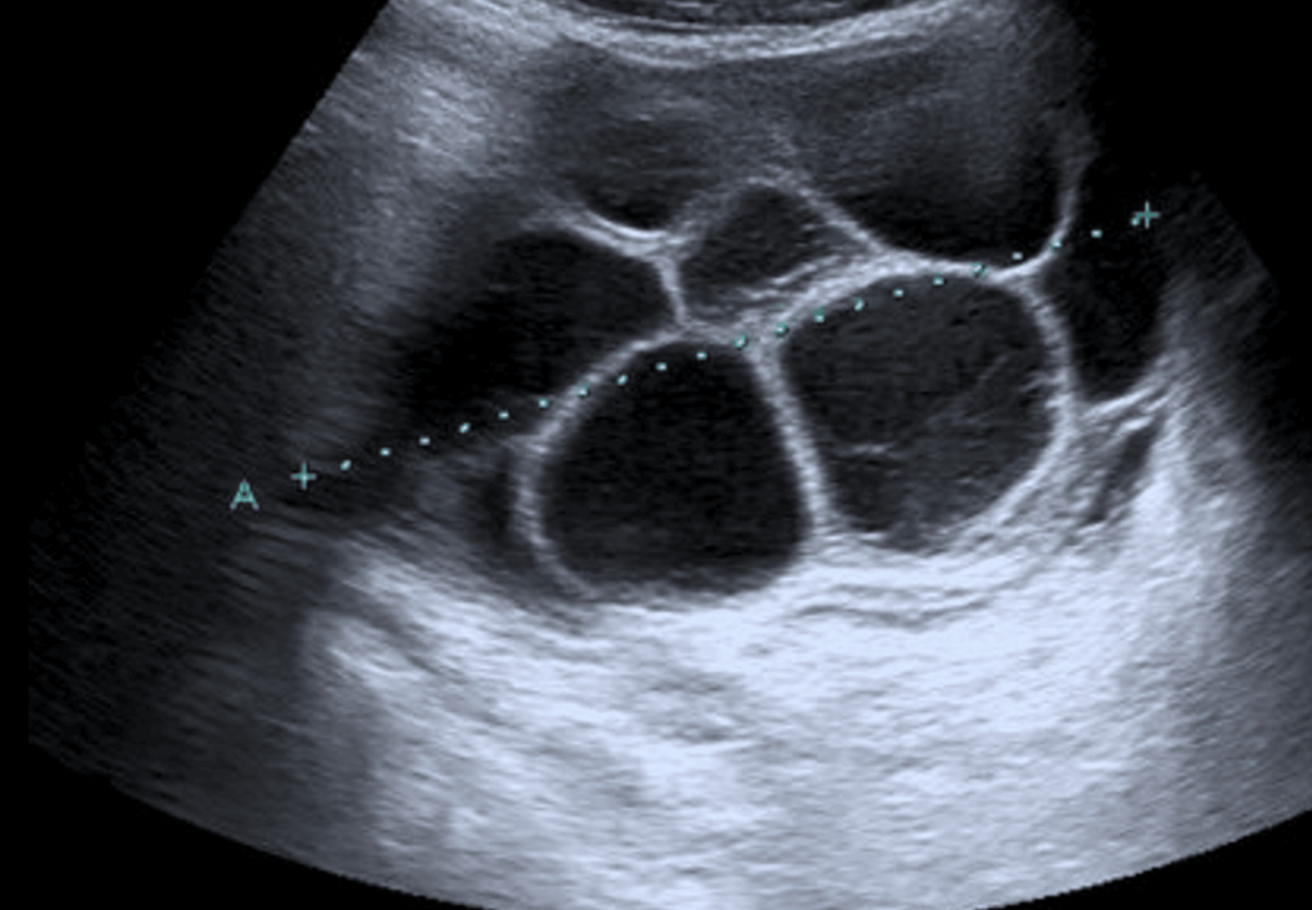
Pelvic ultrasound demonstrating bilateral enlarged multicystic ovaries with thin-walled cysts. Findings are characteristic of ovarian hyperstimulation syndrome in early pregnancy.

Pelvic ultrasonography demonstrated a viable intrauterine pregnancy with a crown-rump length corresponding to seven weeks and two days gestation, with cardiac activity present. Bilateral enlarged multicystic ovaries were noted, measuring 10.2 cm on the right and 9.8 cm on the left, with multiple thin-walled cysts (Figure 1). There was also moderate to large free fluid in the pelvis and upper abdomen, consistent with ascites. Chest radiograph revealed bilateral small pleural effusions without overt pulmonary edema.

Given the clinical presentation and investigations, the differential diagnosis included OHSS, ovarian torsion, ruptured ovarian cyst, and, less likely, malignancy such as a germ cell tumor, given the bilateral ovarian enlargement. However, the absence of a history of ovulation induction or fertility treatment initially raised doubts regarding OHSS. Nonetheless, spontaneous OHSS triggered by endogenous hCG in early pregnancy was considered the most likely diagnosis based on clinical, laboratory, and imaging findings.

A definitive diagnosis of spontaneous moderate-to-severe OHSS was made. The patient was admitted to the high-dependency unit for close monitoring. Initial management included aggressive intravenous fluid resuscitation with isotonic crystalloids, careful monitoring of fluid balance, and prophylactic low molecular weight heparin to prevent thromboembolic complications. Electrolyte abnormalities were corrected with targeted therapy. Serial hematocrit, electrolytes, renal function tests, and ultrasonography were performed to monitor the course of the disease. Paracentesis was deferred initially as her respiratory status was stable and abdominal symptoms, though significant, were manageable with conservative measures.

Over the next 48 hours, the patient's respiratory symptoms worsened with increasing oxygen requirements. Repeat ultrasonography showed further ascitic fluid accumulation. Therapeutic paracentesis was performed under ultrasound guidance, yielding approximately 1.2 liters of straw-colored fluid, which led to symptomatic improvement in abdominal distension and respiratory status. Fluid cytology was negative for malignancy. The patient remained hemodynamically stable, and there was no evidence of ovarian torsion or rupture on follow-up imaging.

Her hospital course was notable for gradual improvement over one week. No thromboembolic events occurred. The patient was transitioned to oral fluids as tolerated and continued to receive prophylactic anticoagulation. By hospital day 10, her hematocrit normalized, and her abdominal girth decreased significantly. She was discharged home with close outpatient follow-up. At her two-week follow-up appointment, she remained clinically well, and ultrasonography showed resolution of ascites with persistent but regressing ovarian enlargement. The intrauterine pregnancy remained viable, and she continued routine antenatal care under obstetric surveillance.

## Discussion

OHSS is a potentially life-threatening condition traditionally linked to exogenous gonadotropin use in assisted reproductive technologies. However, spontaneous OHSS, though rare, presents unique diagnostic and management challenges, particularly in spontaneous pregnancies without preceding fertility treatment. The present case highlights the importance of maintaining a high index of suspicion for OHSS in pregnant patients presenting with ascites, pleural effusions, and ovarian enlargement, even in the absence of ovulation induction therapy.

Spontaneous OHSS is hypothesized to result from an exaggerated ovarian response to endogenous hCG, leading to increased VEGF expression and subsequent vascular permeability. The syndrome has been classified into three categories based on underlying etiology: spontaneous OHSS associated with multiple gestations or molar pregnancies with high hCG levels, spontaneous OHSS linked to hypothyroidism, and a rare form associated with activating mutations of the follicle-stimulating hormone receptor [5,6]. In the present case, a singleton intrauterine pregnancy was confirmed, and thyroid function tests were normal, suggesting a primary exaggerated ovarian sensitivity to physiological hCG levels rather than hCG excess or thyroid dysfunction.

The clinical presentation of spontaneous OHSS mirrors that of iatrogenic forms, characterized by hemoconcentration, ascites, electrolyte imbalance, and risk of thromboembolism. In our patient, moderate-to-severe OHSS developed, necessitating hospitalization, supportive care, and therapeutic paracentesis. Notably, the absence of prior fertility treatment delayed the initial consideration of OHSS in the differential diagnosis, underscoring a frequent pitfall in clinical recognition [3,7]. Early diagnosis is crucial, as delays may increase the risk of complications such as renal impairment, venous thromboembolism, and respiratory compromise due to massive effusions.

Management of spontaneous OHSS is largely supportive and mirrors that of the iatrogenic form, focusing on fluid resuscitation, correction of electrolyte disturbances, thromboprophylaxis, and paracentesis for significant ascites or pleural effusions. The decision to intervene invasively must be carefully weighed against potential risks, particularly in early pregnancy [3-7]. Our patient's favorable response to conservative management with adjunctive therapeutic paracentesis highlights the importance of individualized care plans based on disease severity and symptomatology. Importantly, there are no evidence-based guidelines specifically tailored to spontaneous OHSS, and management recommendations are extrapolated from experience with iatrogenic cases.

Pregnancy outcomes following spontaneous OHSS are generally favorable with appropriate management, although the risk of miscarriage and pregnancy complications such as preeclampsia may be slightly increased [8,9]. In our case, the ongoing viability of the pregnancy was maintained, and the patient experienced a gradual resolution of symptoms. Serial imaging demonstrated regression of ovarian enlargement, consistent with the natural course of OHSS as hCG levels plateau and subsequently decline in later pregnancy.

## Conclusions

In conclusion, spontaneous OHSS, though rare, is an important differential diagnosis in early pregnancy, particularly in patients presenting with ascites, pleural effusions, and ovarian enlargement without a history of fertility treatment. Early recognition and appropriate supportive management are essential to prevent serious maternal and fetal complications. This case underscores the need for heightened clinical awareness and highlights the importance of considering spontaneous OHSS even in singleton pregnancies with physiological hCG levels. Ongoing reporting and analysis of such cases are vital to deepen understanding of their pathophysiology and to guide future evidence-based management strategies.
